# Size matters

**DOI:** 10.1186/bcr2989

**Published:** 2011-11-04

**Authors:** M Powell, B Bayley

**Affiliations:** 1 Breast Test Wales, Wrexham, UK

## Introduction

It is accepted that a proportion of patients treated by breast-conservation surgery for unifocal malignancy will undergo further surgery for residual disease. Preoperative planning for impalpable lesions involves a radiological prediction of the extent of disease. The optimal outcome is disease clearance achieved by one operation only. We have revisited our cases which required a subsequent theatre visit in order to facilitate an understanding of the factors involved in accurate lesion sizing and thereby reduce re-excision rates.

## Methods

All women screened through the Wrexham centre with a positive diagnosis of *in situ *or invasive disease going on to have operative treatment over a 1-year period have been included. The lesions were double-read by experienced film readers to obtain a maximal dimension on two-view mammography and ultrasound where applicable. Each case was attributed a grading, 1 to 3, for ease of measurement. This was compared with the postoperative histology, reported by a specialist breast histopathologist. The size, type, grade, receptor status and nodal status were recorded for each case.

## Results

A total of 61 cases were selected. The preoperative prediction of size was closest to the mammographic measurement in 74% of cases, and to the ultrasound dimension in 21%. The remainder showed equal measurements on both.

## Conclusion

The mammogram provides a more accurate prediction of lesion size when compared with ultrasound images. It should be borne in mind that a certain proportion of women will choose mastectomy over conservation regardless of the available option of conservation.

